# Adaptive evolution of the lower jaw dentition in Mexican tetra (*Astyanax mexicanus*)

**DOI:** 10.1186/2041-9139-4-28

**Published:** 2013-10-07

**Authors:** Atukorallaya Devi Sewvandini Atukorala, Christine Hammer, Megan Dufton, Tamara Anne Franz-Odendaal

**Affiliations:** 1 Department of Biology, Mount Saint Vincent University, 166 Bedford Highway, Halifax, Nova Scotia B3M3J8, Canada; 2 Department of Biology, Dalhousie University, 1355 Oxford Street, Halifax, Nova Scotia B3H4R2, Canada

**Keywords:** Cavefish, Oral jaws, Mandible, Premaxilla, Maxilla, Tooth, Cusps

## Abstract

**Background:**

The Mexican tetra (*Astyanax mexicanus*) has emerged as a good animal model to study the constructive and regressive changes associated with living in cave environments, as both the ancestral sighted morph and the cave dwelling morph are extant. The cave dwelling morphs lack eyes and body pigmentation, but have well developed oral and sensory systems that are essential for survival in dark environments. The cave forms and surface forms are interfertile and give rise to F1 hybrids progeny known as intermediates. In cavefish, degeneration of the lens is one of the key events leading to eye regression. We have previously shown that surgical lens removal in surface fish embryos has an effect on the craniofacial skeleton. Surprisingly, lens removal was also found to have an effect on the caudal teeth in the lower jaw. In order to understand this result, we analyzed the lower jaw and upper jaw dentitions of surface, cavefish and F1 hybrids of surface and cavefish and compared our findings with surface fish that underwent lens removal. We also investigated the upper jaw (premaxillae and maxillae) dentition in these fish.

**Results:**

Our tooth analyses shows that cavefish have the highest numbers of teeth in the mandible and maxillae, surface forms have the lowest numbers and F1 hybrids are between these groups. These differences are not observed in the premaxillae. A wide diversity of cuspal morphology can also be found in these fish. Jaw size also differs amongst the groups, with the mandible exhibiting the greatest differences. Interestingly, tooth number in surgery fish is different only in the caudal region of the mandible; this is the region that is constrained in size in all morphs.

**Conclusion:**

Our data provides the first detailed description of the jaw dentitions of two morphs of *Astyanax mexicanus*, as well as in F1 hybrids. Tooth number, patterning and cuspal morphology are enhanced in cavefish in all jaws. This is in contrast to the increase in tooth number previously observed on the lens ablated side of the surgery fish. These findings indicate that the mechanisms which govern the constructive traits in cavefish are different to the mechanisms causing an increase tooth number in surgery fish.

## Background

Over the course of vertebrate evolution organisms have adopted constructive and regressive changes favorable for their habitats. The creatures living in the extreme environments, for instance cave dwelling animals, are popular organisms to study because of their wide variety of adaptive features. Thus great insight can be gained when comparing cave-adapted animals to their close relatives who do not live in these environments.

In recent years, the teleost fish Mexican tetra, *Astyanax mexicanus*, has been identified as a useful model for studying the evolutionary biology of eye development. This is a single species consisting of two morphological types, an eyed and pigmented surface-dwelling form (surface fish) and an eyeless unpigmented cave-dwelling (cavefish) form [1-5]. These cavefish diverged from their surface fish ancestors, probably about 1 million years ago, and approximately 29 different cavefish populations have been identified [1-3,6]. The two morphs can easily interbreed giving rise to intermediates.

The most highly investigated feature of the cavefish is their lack of eyes in adulthood. Despite the fact that cavefish do not have eyes as adults, eye development begins in the same manner in both the surface and cavefish morphs up until 24 hours postfertilization (hpf) [7,8]. At this age, the eye starts to degenerate. In addition to eye degeneration, cavefish have other regressive changes including loss of pigmentation, reduction in the size of the optic tectum, and a reduction in aggressive and schooling behavior [1,2,5,9]. Less commonly studied are the constructive changes, which include changes in body position while feeding, increased size and number of cranial neuromasts, larger fat stores, increased number of taste buds, increased number of teeth and increased jaw size [2,5,9].

Although the increased tooth number in cave forms is generally accepted, only one study has investigated tooth differences between cavefish and surface fish and it largely focused on teeth of only one oral jaw, namely the maxillary bone [10]. In addition, there are no data on tooth patterning in F1 hybrids of cavefish and surface fish crosses, which are commonly known as intermediates. The Mexican tetra bears teeth on the premaxilla, maxillary, and dentary bones of the oral jaws and on the gill rakers, upper pharyngeal tooth plates and the fifth ceratobranchials of the pharyngeal skeleton [11]. The unicuspid larval dentition is gradually replaced by the bicuspid then tricuspid teeth resulting in the oral adult dentition. Generally the dentary bone of the adult surface morphs bears eight large multicuspid teeth rostrally and several small multicuspid and unicuspid teeth caudally [11]. The diverse cuspal morphology can be found in the rostral teeth, which varies from five to eight cusps. During food acquisition the lower jaws occlude with the upper jaws (the premaxillae and maxillae). Premaxillary teeth directly contact the mandibular teeth while the maxillary teeth are positioned 180° to the functional position. The premaxilla has two rows of teeth with seven to nine teeth per side of the jaw while each maxilla bears none, one or two teeth on each side [11]. Cuspal morphology in the upper jaw teeth are similar as described above for the mandible. These teeth help the fish to grasp and chew food; nearly 40 tooth replacement cycles occur throughout the life of the surface morph [11].

**Figure 1:**
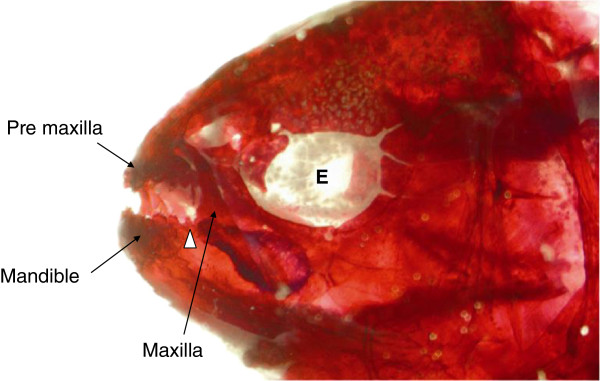
**Lateral view of the surgery side of the 3 days postfertilization surgery fish.** Alizarin red stained and cleared adult *Astyanax mexicanus* surface fish which has undergone surgical removal of the lens at 3 days postfertilization. The surgery eye is indicated by E. White arrowhead indicates the caudal region of the mandible.

We previously demonstrated that, by removing the lens of one eye at several time points in surface tetra embryos, lens removal has an effect on tooth development of the caudal region of the mandible (Figure 1) [12]. An increased number of caudal teeth were found on the surgery side compared to the non-surgery side. Generally, caudal teeth are susceptible to natural variation making them more plastic in nature [11]. Their small size, the timing and manner of their development, and their location close to the optic cup might make these teeth more susceptible to influences from the developing eye than the central larger multicuspid teeth.

Furthermore, we determined that the surgery fish are a useful group in which to be begin to unravel the transition from surface to cavefish [12].

To date, a detailed comparison of the characteristics of the lower jaw dentition in the *Astyanax* system has not been conducted. In this present study, we analyze and describe the tooth morphology, patterning, cuspal morphology and jaw dimensions in surface, cavefish and their F1 hybrids. For a more comprehensive analysis, and since the mandible occludes with the upper jaws, we include both premaxillae and maxillae in our analysis. Finally we compared the above results with the surgery-induced jaw phenotypes, which we described in our previous study [12] in order to fully understand the gross morphological diversity of the oral jaws associated with eye regression in *Astyanax mexicanus*.

## Methods

### Material

Surface Mexican tetra (*Astyanax mexicanus*) adults were maintained at 21°C on a 12-hour light, 12-hour dark cycle. To induce spawning, tank temperature was increased to 26°C and two males were added to a tank containing one female. Eggs were collected the next day. Fish were housed at Mount Saint Vincent University, Halifax Nova Scotia, Canada. Animals were raised according to the guidelines of Canadian Council of Animal Care. All protocols were approved annually.

Tinaja cavefish and F1 hybrids of Tinaja cavefish and surface fish were received in 10% neutral buffered formalin from Dr R Borowsky (New York University, New York City, USA). These Tinaja cavefish were second generation from wild populations.

We examined a total of 27 adult fish: Tinaja cavefish (n=8, 4.08 ± 0.29 cm Standard Length, age 6 years old), F1 hybrid fish (n=6, 5.1 ± 0.17 cm Standard Length, age 7 years old), surface fish (n=8, 4.4 ± 0.3 cm Standard Length , age 3.5± 1.4), and surgery fish (unilateral lens ablation of surface fish embryo at three days postfertilization (dpf) as described in [12]; n=5, 3.8 ± 0.3 cm Standard Length, age 1.0± 2.1). Surgery side and non-surgery side of the surgery fish were considered as two separate groups in most of the analysis giving a total of five groups; when four groups are compared, these are cavefish, F1 hybrids surface and surgery fish. Averages and standard deviations were also calculated.

### Whole mount bone stain

Adult surface fish (n=8) specimens were anesthetized using 0.1% MS222, and then fixed in 10% neutral buffered formalin (23-245-685; Fisher Scientific, Ottawa, Ontario, Canada). Alizarin red (A5533; Sigma-Aldrich Canada Co, Oakville, Ontario, Canada) was used to bone stain the skeletons of surface, cavefish and intermediate fish according to standard protocols. Surgery fish were already stained in our previous study (Figure 1) [12]. Briefly, fish were bleached overnight in 3% hydrogen peroxide in 1% potassium hydroxide (Sigma 1767) solution.The following day, fish were rinsed in water, and then soaked in saturated sodium tetraborate (Sigma B9876) for 8 hours. Fish were stained overnight in Alizarin stain (1mg/ml Alizarin in 1% potassium hydroxide). Finally, specimens were rinsed in 1% potassium hydroxide then cleared in 1% Trypsin (Fisher Scientific, 9002-07-7) and 2% sodium tetraborate in distilled water for 3 nights. The specimens were processed through an ascending series of glycerol in 1% potassium hydroxide solution then transferred to a storage solution of 100% glycerol.

### Counting of teeth and jaw measurements

**Figure 2:**
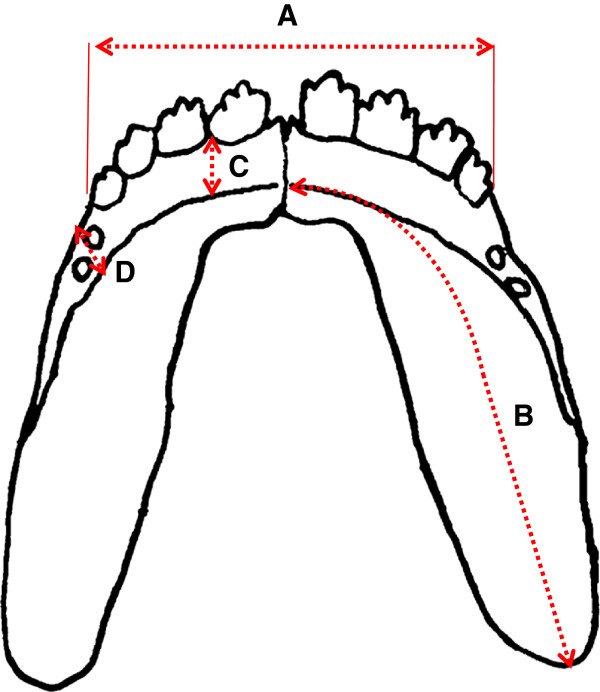
**Schematic diagram of the Mexcian tetra mandible.** The dotted line indicates the measurements taken. **(A)** maximum rostral width, **(B)** mandibular length, **(C)** maximum bone width of the rostral tooth bearing area, and **(D)** maximum bone width of the caudal tooth bearing area.

The mandibles, premaxillae and maxillae were dissected from the above samples in order to accurately measure the jaws and count teeth. For clarity, in our analysis we consider the surgery side as the right side and the non-surgery side as the left side of the fish. Jaws were examined under a Nikon SMZ 1500 stereomicroscope (Kawasaki, Japan). All the teeth of each jaw were counted. Cusp number and tooth position were also noted. The measurements were taken using Nikon NIS Elements software. The following measurements were taken for the mandible: total rostral width, length of one side of jaw, width across the rostral tooth bearing area and caudal tooth bearing area (Figure 2). For the premaxillary bone, we measured the length of one side of jaw, the width across the rostral tooth bearing area and caudal tooth bearing region. For the maxillary bone, we measured the total length and maximum width across the tooth bearing region. Averages and standard deviations were calculated.

One-tailed paired *t* tests and one-way analysis of variance (ANOVA) and multiple comparison Tukey's test were performed using Minitab, version 16, (Minitab Inc, Pennsylvania, USA) and statistically significant outcomes reported as *P* <0.05 in all analyses.

## Results

### The adult lower jaw dentition in *Astyanax mexicanus* morphs

**Figure 3:**
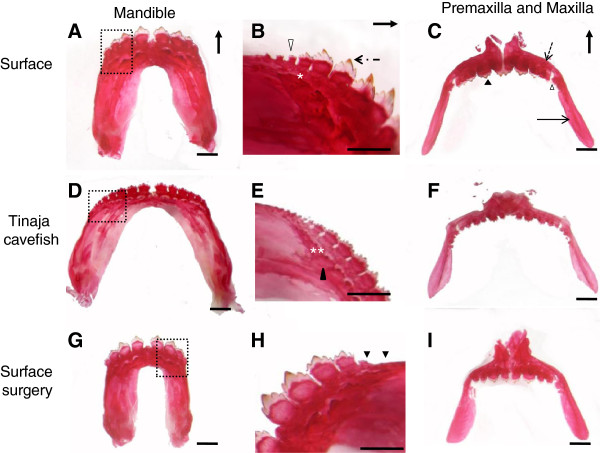
**Dorsal view of the mandible and rostral view of the premaxilla and maxilla of *Astyanax mexicanus*.** Alizarin red stained adult surface fish **(A-C)**, Tinaja cavefish **(D-F)** and surface surgery fish **(G-I)**. **(A,D,G)** show the mandibles. The dotted box in **(A,D,G)** is enlarged in **(B,E,H)**. In **(A,D,G)** teeth are arranged in one row in the rostral rim of the mandible. Rostral teeth are multicuspid and large in size. The black dotted arrow in **(B)** indicates the cusp tip of a multicuspid tooth which is yellow in color due to the iron deposition. The white arrowheads in **(B)** and black arrowheads in **(H)** indicate the caudal teeth which are smaller in size compared to the rostral teeth. The white asterisk in **(B)** indicates the tooth free zone that can be found between the rostral and caudal teeth of the surface fish but which cannot be seen in cavefish (double white asterisk in **(E)**). The black arrowhead in **(E)** indicates the successional tooth row. **(C,F,I)** show the right and left premaxillae and maxillae. In **(C)** premaxilla is indicated by the black dotted arrow and premaxillary tooth is indicated by the black arrowhead. The maxilla is indicated by the black arrow and maxillary teeth are indicated by the white arrow head in **(C)**. The black arrow in the upper right corner of **(A)** and **(B)** indicates the rostral direction. The black arrow in **(C)** indicates the dorsal direction. All scale bars are 100 μm.

The basic skeletal architecture described for the surface fish mandible is similar in cavefish, F1 hybrids and surface morphs [13]. Briefly, the left and right dentaries are attached in the midline by the symphysis and function as a strong unit. Each dentary articulates dorsoposteriorly with the anguloarticular and the retroarticular and posteromedially with coromeckelin bone. The dentary bears a single row of teeth (Figure 3A,D,G). There are four rostral teeth followed by several caudal teeth in the lower jaw for each group examined. The largest rostral tooth is always located more medially and the teeth decrease in size towards the caudal end (Figure 3B,E,H). Each tooth row extends from the mandibular symphysis to the caudal region of the mandible (Figure 3A,D,G). In the caudal region, the teeth are much smaller (Figure 3B,H, arrowhead) and tooth positioning does not follow a strict linear pattern (or line) of eruption (described below).

First we describe the tooth number, pattern and morphology of mandibular teeth in each group then we discuss jaw measurements. Finally, we discuss the premaxillary and maxillary jaws.

### Patterning of the lower jaw dentition

In all fish examined, the teeth in the mandible are arranged in a single row. The four large multicuspid teeth are tightly packed at the rostral edge of the mandible. There is a small tooth-free zone present between the rostral teeth and caudal teeth (Figure 3B, white asterisk) in surface and surgery fish. This zone is not present in most of the F1 hybrids and cavefish (Figure 3E, double white asterisk). In general, rostral teeth point rostrodorsally while caudal teeth point medioventrally. The row of successional teeth is always positioned labial to its functional tooth (Figure 3E, black arrowhead).

In all four groups we examined, tooth arrangement is haphazard in the caudal region of the jaw. Some caudal teeth can be found towards the labial side of the mandible while in some groups these teeth are positioned lateral to the rostral teeth.

### Mandibular tooth numbers

Although, multicuspid tooth number is constant in each group (four teeth per dentary), the caudal teeth are highly variable in number. Tinaja cavefish have on average 12 ± 2 teeth in each side of the mandible while surface fish have an average of 8 ± 1 teeth (Additional file 1: Figure S1). F1 hybrid fish have 10 ± 1 teeth in the mandible (Additional file 1: Figure S1). The surgery side of surface fish have an average of 7 ± 0.89 teeth, while the non-surgery side of the surgery fish jaw has 5 ± 1.14 teeth. Total tooth number in the mandible differed significantly among the groups (one-way ANOVA, *P* <0.05, F (5.27)),with cavefish having the most teeth and surgery fish the least. Multiple comparison testing shows the following comparisons are significantly different: Surface fish–cavefish; surface fish–F1 hybrids; cavefish–surgery side of the surgery fish; F1 hybrids–surgery side of the surgery fish; cavefish–non-surgery side of the surgery fish; F1 hybrids–non-surgery side of the surgery fish. Importantly, there is no difference between the surface and surgery forms. The left and right sides of surface fish, cavefish and F1 hybrids are not statistically significant with respect to tooth number (Additional file 1: Figure S1), surface fish: paired t-test, *P* > 0.36, F=8; cavefish: paired t-test, *P* >0.22, F=8; F1 hybrids: paired t-test, *P* >0.5, F= 6). However, the left (non surgery side, control) and the right (surgery) halves of the mandible of surgery fish are statistically significant (paired t-test, *P* <0.05, F=4) with the non-surgery side having fewer teeth (Additional file 1: Figure S1). Interestingly one surgery fish had no caudal teeth on the non-surgery side and three teeth on the surgery side.

### Morphology of mandibular teeth

A large variation in tooth size and cusp number was also observed (Table 1). Despite this, all teeth are attached to the bone with one root and teeth have between one and eight cusps (Table 1).These cusps are conical and pointed, they are positioned mesiolaterally, and the enameloid cap shows iron deposition (Figure 3B, dotted black arrow).

**Table 1:** Tooth and cusp numbers in the rostral and caudal regions of the mandible for different morphs of the Mexican tetra

**Group**	**SL (cm)**	**Total tooth number**	**Rostral cusp number**					**Caudal tooth number**	**Caudal cusp number**						
			**4 cusps**	**5 cusps**	**6 cusps**	**7 cusps**	**8 cusps**		**1 cusps**	**2 cusps**	**3 cusps**	**4 cusps**	**5 cusps**	**6 cusps**	**7 cusps**
C-1	5.7	7		3	1			5				1	1	1	
C-2	4.7	9	1	3				5	2	1	1	1			
C-3	4.7	7		4				3	2			1			
C-4	5.3	6		4				2			1	1			
C-5	5.5	6		4				2			1	1			
C-6	5.7	9		4				5			4			1	
C-7	4.6	9		4				5	2		3				
C-8	4.0	10		4				6	1	2	2	1			
**Average number of teeth**		**7.88 ± 1.55**						**3.88 ± 1.55**							
**Average cusp/tooth**		**4.04**						**2.87**							
S-1	3.7	7		2				3	2		1				
S-2	4.1	7		4				3			2	1			
S-3	4.3	7		4				3			1	2			
S-4	3.6	7		4				3			1	2			
S-5	3.7	9		4				5			3		2		
**Average number of teeth**		**7.4 ± 0.89**						**3.4 ± 0.89**							
**Average cusp/tooth**		**4.26**						**3.29**							
Non Sx-1	3.7	4		4				0							
Non Sx-2	4.1	6		4				2	1			1			
Non Sx-3	4.3	6		4				2			1	1			
Non Sx-4	3.6	7		4				3			1	1			
Non Sx-5	3.7	8		4				4			2	1			
**Average number of teeth**		**6.2 ± 1.48**						**2.2 ± 1.48**							
**Average cusp/tooth**		**4.84**						**3.29**							
T-1	3.7	11		1	1	2		7		2	3	1	1		
T-2	4.7	12			3	1		8	1	1	4		1	1	
T-3	4.7	13		2	2			9	1	2	4		2		
T-4	4.0	10			3	1		6	1	1	2	1	1		
T-5	5.0	14			3	1		10		4	4		1		1
T-6	7.1	14			2	2		10	1		4	1	2	2	
T-7	4.4	8		1	2	1		4	1	1				1	
T-8	4.2	11			2	1	1	7		2	4			1	
**Average number of teeth**		**11.62 ± 2.06**						**7.62 ± 2.06**							
**Average cusp/tooth**		**4.16**						**3.18**							
I-1	4.9	10			3	1		6	1	2	2		1		
I-2	5.7	12			1	3		8		1	4	1	1	1	
I-3	5.8	13			3	1		9		1	5		3		
I-4	5.2	8			4			4	1			1	1	1	
I-5	5.4	11		1	2	1		7		1	4			2	
I-6	5.2	10			4			6			3	1	2		
**Average number of teeth**		**10.67 ± 1.75**						**6.67 ± 1.75**							
**Average cusp/tooth**		**4.56**						**3.55**							

C-1 to C-8, surface control fish; S-1 to S-5,surface surgery fish, surgery side; NonSx-1 to Non Sx-5, surface surgery fish non-surgery side; T-1 to T-8, Tinaja cavefish; I-1 to I-6, intermediates (F1 hybrids). The rostral teeth cusp number varies between four to eight cusps while the caudal teeth cusps number varies from one to seven cusps per tooth. Averages ± standard deviations are given. Standard Length (SL).

The rostral teeth are large, multicuspid, short teeth. In this set of teeth, the third tooth is always similar in size to the first tooth. Tooth sockets for each rostral tooth are well developed and teeth are firmly attached to the mandible by one root. This general pattern was observed in surface fish, cavefish, F1 hybrid fish and in surgery fish. Quantitative analysis revealed that cusp number varies within a range of four to eight cusps per rostral tooth (Table 1). Cavefish have the most cusps, followed by F1 hybrids and surface fish. The surface and surgery fish have on average five cusps per rostral tooth, while the cave and F1 hybrids have six or seven cusps per rostral tooth (Table 1).

The caudal teeth are short and small in size compared to rostral teeth (Figure 3B,H arrowhead). These teeth are also attached to the bone but the most caudally positioned tooth sockets are not as deep as those of the rostral teeth. For example, some caudal teeth are large and unicuspid while others are small and multicuspid (Figure 3B, arrowhead). Further, we also calculated the average cusp number per tooth in each group by dividing the total number of cusps by the tooth number (Table 1). The cusps/tooth ratio was not significant across the five morphs (one-way ANOVA, *P* >0.05, F (4.27)). The paired *t* test showed that there is no difference in cusp number in surgery versus non-surgery sides of the surface fish mandible (paired *t* test, *P* >0.5, F= 4, Table 1).

### Diversity of the mandible - shape and size

In order to determine the morphological diversity of the dentary bone associated with the different eye and tooth phenotypes, we took several measurements of the mandible (Figure 2) in each of the five groups (cavefish, F1 hybrids, surface fish, surgery side of the surgery fish and non-surgery side of the surgery fish).

The width across the rostral tooth bearing area (distance A in Figure 2) is significantly different across groups (Table 2). The length of each side of the mandible (distance B in Figure 2) and the width of the rostral tooth bearing bone (distance C in Figure 2) was statistically different across the groups; however, the width of the caudal tooth bearing region (distance D in Figure 2) was not significantly different amongst the groups (Table 2). Furthermore, the surgery side and the non-surgery side was not statistically different with regard to the rostral and caudal bone width (paired *t* test, *P* >0.5, F= 4).

**Table 2:** The mandibular measurements of surface control, Tinaja cavefish, F1 hybrids and surgery and non-surgery sides of the surgery fish

**Group**	**Rostral width (Distance A)**	**Side length (Distance B)**	**Rostral bone width (Distance C)**	**Caudal bone width(Distance A)**
Surface (n = 8)	3.07 ± 0.56	4.03 ± 0.47	0.76 ± 0.13	0.25 ± 0.07
Tinaja Cavefish (n = 8)	4.20 ± 0.98	4.38 ± 0.77	0.68 ± 0.91	0.21 ± 0.04
F1 hybrids (n = 6)	4.08 ± 0.65	4.29 ± 0.65	0.84 ± 0.11	0.19 ± 0.12
Surgery surgery side (n=5)	3.00 ± 0.64	3.43 ± 0.36	0.62 ± 0.07	0.23 ± 0.12
Surgery non-surgery side (n=5)		3.23 ± 0.56	0.66 ± 0.07	0.23 ± 0.09
Statistical analysis	*P* <0.05	*P* <0.05	*P* <0.05	*P* <0.05
(one way ANOVA)				
Tukey HSD	Surface and F1-hybrids, surgery and F1-hybrids	Surface and surgery side, surface and non-surgery side, surgery side and F1-hybrids, surgery side and cavefish, F1-hybrids and non-surgery side, cavefish and non-surgery side	F1-hybrids and cavefish	none
95% significance				
			Cavefish and non-surgery side	

See Figure 2 for details of each measurement. Measurements are in mm. Averages ± standard deviations are given. ANOVA, analysis of variance; HSD honest significant difference.

In summary, the mandible has different dimensions in each group except in the caudal region, where caudal width appears to be constrained. This is particularly interesting, given that a large difference in tooth number was observed in this region of the mandible after the surgery [12].

### The upper jaws - premaxillae and maxillae

During food acquisition, the premaxillary teeth occlude with the mandibular teeth while the maxillary teeth are positioned at 180° to the mandible (Figure 1). In order to compare the lower jaw phenotypes with the upper jaws we examined the tooth bearing premaxillae and maxillae. Similar to the mandible, the basic skeletal architecture of the above bones are similar across different morphs [13]. The premaxilla is a triangular shaped bone (Figure 3C,F,I). The left and right premaxilla are attached dorsally with the ethmoid and nasal bones, and laterally with the maxilla (Figure 1). Each premaxillary bone has two rows of teeth which run rostro-caudally. Typically the anterior row has four to five teeth while the posterior row has five to seven teeth. The teeth are large, multicuspid and short (Figure 3C, black arrowhead). Tooth sockets are similar to the mandible and are firmly attached to the bone by one root. This general pattern was observed in surface fish, cavefish and their F1 hybrids and in surgery fish.

**Figure 4:**
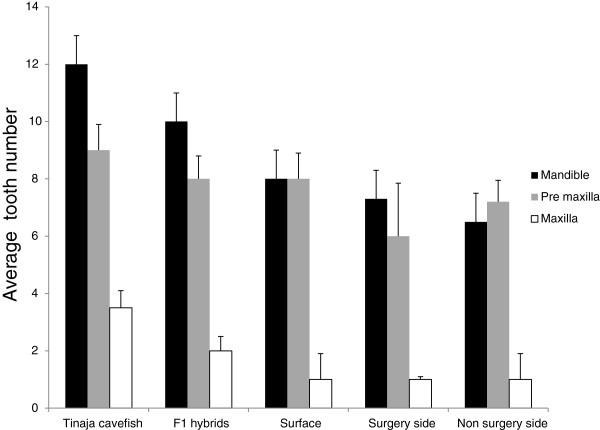
**Bar graph showing the average number of teeth in the mandible, premaxilla and maxilla of surface, Tinaja cavefish, F1 hybrids, and surgery surface fish.** Error bars indicate standard deviation.

The total tooth number in each premaxilla among the morphs is as follows (Figure 4): Tinaja cavefish have on average 9 ± 0.9 teeth, F1 hybrids have 8 ± 0.8 teeth, while surface fish have average of 8 ± 0.9 teeth. The surgery fish have 6.6 ± 1.85 teeth on the surgery side and 7.2 ±0.75 teeth on the non-surgery side. Total tooth number in the premaxilla is not significantly different among the five groups (one-way ANOVA, *P* >0.05, F (4.27)). Slightly higher tooth numbers could be observed in the cavefish premaxilla. Unlike the mandible, there were not any statistical differences in tooth number between the surgery and the non-surgery side of the premaxilla (paired *t* test, *P* = 0.18, F=4) (Figure 4). Using the same method of calculation previously described for the mandible, we determined the cusp/tooth ratio for each side of the premaxilla; these are not statistically different among the five groups (one-way ANOVA, *P* >0.05, F (4.27).

Furthermore, there is no significant difference in the length of the each premaxilla amongst the groups (one-way ANOVA, *P* >0.05, F (4.27)). Interestingly the maximum width of the rostral tooth bearing area and the maximum width of the caudal tooth bearing area is significantly different among the groups (one-way ANOVA, *P*< 0.05, F (4.27)). Multiple comparison tests show the following comparisons are significantly different: surface–cavefish, surface–F1 hybrids. This suggests that cavefish and F1 hybrids have wider premaxillae than surface forms.

The maxillary bone is long and thin, articulating dorsally with the premaxilla (Figure 1, Figure 3C,F,I). The posterior ramus of maxilla overlaps the dentary. The maxillary teeth which always develop intraosseously (Figure 3C, white arrowhead) attach to the bone by one strong root and the cusp number varies between four and six. These cusps are always positioned 180° to the functional position of the jaws.

Tooth numbers in the maxillary bone varies across the four morphs as follows (Figure 4): Tinaja cavefish have on average of 3.54 ± 0.5 teeth on each maxillary bone, F1 hybrids have 2 ± 0.6 teeth, while surface fish have an average of 1 ± 0.9 teeth. The surgery side of the maxilla has 1 ± 0.1 teeth while the non-surgery side maxilla has 1 ± 0.9 teeth. The maxillary tooth number is different across morphs (one way ANOVA *P* < 0.05, F (4.27)). Multiple comparison shows the following comparisons are significantly different: surface fish–cavefish, surface fish–F1 hybrids, cavefish–F1 hybrids, but there is no difference between surface and surgery forms. Furthermore, paired *t* test shows that there is no significant difference in the tooth number in the surgery side versus the non-surgery side of the maxilla (paired *t* test, *P* = 0.18, F=4) (Figure 4).

The length and width of the maxillae were found to be statistically significant across the groups (one-way ANOVA, *P* < 0.05, F (4.27)). Multiple comparison testing shows that this difference is significant in the following comparisons: surface–cavefish, surface–F1 hybrids, F1 hybrids–surgery side of the surgery fish, cavefish–surgery side of the surgery fish, and non-surgery side of the surgery fish–F1 hybrid fish.

In summary, tooth number does not differ significantly in the premaxillae; however, slightly higher tooth numbers were noted in the cavefish. In the maxillae, tooth number differs across the morphs. The width of the premaxillae and the length and width of the maxillary bone is different across the four groups examined.

## Discussion

After diverging from their ancestral surface fish, the blind cavefish has thrived for nearly 1 million years in a food sparse, perpetual dark environment. While regressive changes such as eye degeneration and loss of pigmentation evolved, so did constructive changes such as enlarged jaws, increased taste buds and increased tooth number [1,2,5,9].

Although much research has been conducted on the loss of eyes and the effect of lens degeneration on associated or surrounding eye structures [2,8,10,12], few studies have focused on the teeth of these animals and those studies were mainly done on Pachón cavefish [10,11]. So far, very little research has focused on the Tinaja cavefish. These cavefish are known to have regressed non-functioning eyes and minimal body pigments during adulthood [2,8]. The intermediate form (F1-hybrids from cavefish and surface cross) phenotypically fall in the middle of these two groups (that is, they have small eyes and a medium amount of body pigment). How the presence of a small eye influences the development of the jaw bones and oral dentition has not been studied before. In this study we compare the variation in the oral jaw dentitions in three populations of *Astyanax mexicanus* (surface fish, Tinaja cavefish and in the offspring of surface and Tinaja cavefish). We then compared these three groups with experimentally lens-ablated surface fish to examine the effect of lens removal on oral jaw dentition (Figure 1).

### A diverse adult dentition in different eye phenotypes

Our observations on the surface tetra lower jaw data agrees with previous descriptions of surface fish lower jaw dentition [11,13]. Rostral teeth are always constant in number (four teeth per half jaw) and multicuspid with four to eight cusps per tooth. This is in contrast to caudal teeth which are variable in number and consist of small multicuspid and unicuspid teeth (Figure 3B,E,H). We found significant differences in the variation in tooth number in the mandible between each group, with cavefish having the most teeth and surgery fish the least (Figure 4). Interestingly, this type of variation is not present in the premaxillary dentition, which occlude with the mandibular teeth (Figure 4). The maxillary tooth number was significantly different across the four groups. The presence or absence of an eye, or reduced eye size, might have a differential effect on each tooth bearing region. The oral region of the teleost skull could be influenced by different factors, so it is difficult to determine whether the absence of the eye is the primary influence on the tooth number in each of these bones.

Before gaining adult dentition, the Mexican tetra usually passes through several tooth replacement cycles. The transition of unicuspid first generation teeth to subsequent multicuspid dentition in oral jaws in tetra may be associated with the differing feeding demands at different life history stages (for example, soft versus hard prey). A study conducted in two cichlids species from Lake Malawi demonstrated that the timing of turnover from first generation to replacement teeth differs among species and is related to their feeding ecology [14]. The hardness of the food may influence the forces applied to the jaw when eating, resulting in changes in jaw shape and tooth morphology over ontogeny. In *Astyanax*, the first generation teeth are always simple and conical in shape and have an extraosseous origin [11]. With subsequent tooth replacements, teeth develop intraosseously (inside the mandible) and their structural morphology becomes more complex with increasing number of cusps [11]. Similarly the bottom feeding behavior of cavefish requires them to position their mouth at a 45° degree angle so that the mouth can sample substrates in cave pools. Thus, an increase in jaw size, which accommodates a large number of teeth, could have evolved as an adaptation to the challenges of searching for and sampling food in a cave environment [3]*.* Our study consists of mature adults; however, the surface fish and surgery fish were younger than the cavefish and F1 hybrids. A close examination of changes in jaw shape over ontogeny would help to resolve these differences.

The length and width of the mandible (Figure 2A,B,) is also largest in the cavefish (Table 2). Wider jaws have been reported in Pachón cavefish [15]. However, in this study, only one measurement of mandibular size was made; this was the maximum caudal width from left to right. This measurement gives no indication of space available for teeth in the caudal region. Here, we determined that the width of the rostral tooth bearing region was significantly different across the morphs (Table 2) whilst the width of the caudal region is constrained. The increase in rostral width in *Astyanax* cavefish may accommodate more teeth such that in addition to the four rostral teeth there are several multicuspid teeth (Figure 3E) anterior to the small caudal teeth; these rostral teeth might aid the food grasping ability of the cavefish in their extreme environment. The length of the mandible (Figure 2B) is also largest in the cavefish followed by their F1 hybrids and surface and surgery forms (Table 2). The finding that the width of the caudal tooth bearing region does not show any significant difference among these four groups (Table 2) is particularly interesting given that both a large difference in tooth number was observed in this region of the mandible across these groups and also that we found increased tooth number after lens ablation [12]. Moreover, the caudal region has more teeth in the cavefish. This suggests that space available for teeth is not the primary factor influencing the number of teeth that develop in teleost jaws.

Even though there is no difference in the length of the premaxilla, we found significant differences in the width of the rostral and caudal tooth bearing area amongst the four groups, with the cavefish and F1 hybrids having the largest jaws.

Despite blindness, constructive traits have armored cavefish for their survival in a dark, food sparse environment. The sensory organs in blind cavefish can be identified as independent, yet interacting modules [16,17]. Modules exist as networks of gene expression, cell types and developmental processes; natural selection may act on modules at any of these levels [16,17]. Dentitions and the oral jaws can be considered as separate modules. A recent study on a cave catfish (*Astroblepus pholeter*) revealed mechanoreceptor action of skin denticles [18]. These teeth-like structures helped *Astroblepus* to detect the water flow in their cave environment. Skin denticles are considered to be serially homologous structures to oral and pharyngeal teeth [19,20]. As an adaptation to the dark cave environment, the oral dentition of cavefish might have evolved as specialized sensory organs. As in most other teleost teeth, tetra teeth must have an innervated pulp cavity [21]. The role of tetra teeth as a sensory organ needs to be investigated. The mandible houses the teeth, and supports associated sensory structures (taste buds and some neuromasts of the lateral line canal system), which can be considered as the most robust jaw in the oral region.

Eye regression seems to have a differential effect on each jaw module. The gene regulatory network underlying these modules seems to be closely interlinked. For instance, the signaling peptide encoded by the Sonic hedgehog (*Shh*) gene was found to play a central role in cavefish eye degeneration [8,15,22]. A study overexpressing *Shh* in surface fish and inhibiting *Shh* in cavefish during early development determined that there was an increase in taste bud number and mandible size (left to right width) with overexpression in surface fish. *Shh*, which can act as a long- and short-range molecule, plays a pivotal role in vertebrate development. A recent study also found that signaling pathways such as *Shh* are responsible for the replacement of teeth and cuspal transition in cichlid fish, and these signals act in a dose-dependent manner to cause these changes [23]. The pleiotropic effect of the *Shh* gene pathway might underlie the increase in tooth number observed in cavefish [15].

### Surgery surface fish mandibular caudal tooth number and jaw shape

The significant increase in caudal tooth number on the lens-removed side of the mandible was described in our previous study [12]. The lens was removed at four different time points. The difference in caudal tooth number was more prominent when the lens was removed at 3 dpf [12]. In the current study, we determined that the surgery side had on average 7 ± 0.89 teeth while the non-surgery side had on average only 5 ± 1.14 teeth. Despite this increase the total tooth number is still less in the lens removal (surgery) group compared to the other three groups (cavefish, F1 hybrids, non-surgery surface fish) examined (Figure 4).

Only one other study has manipulated the eye and examined tooth number. In that study, the embryonic lens was transplanted between the surface and Pachón cavefish and maxillary tooth number was examined [10]. These authors showed that maxillary tooth number was not affected and they concluded that the maxillary teeth are not influenced by a transplanted cavefish lens. They did not, however, examine the other jaw bones.

We also did not find a significant difference in tooth number in the surgery side and non-surgery side of the premaxillae and maxillae (Figure 4). The effect of lens removal seems to have a direct effect on the tooth development at the caudal part of the mandible, but not on upper jaws, and not on the size of the tooth-bearing region. This may be due to the developmental differences between the jaw teeth in Mexican tetra (age at development and different replacement cycles).

Furthermore, we found that the relative proportion of rostral width and side length of the mandible is similar for cavefish, F1 hybrids and surface surgery fish (Table 2), giving the mandible a somewhat square shape. In our previous study, we applied a limited number of landmarks (that is, 11) for the morphometric analysis of jaw shape. We compared surgery side with the non-surgery side and found that there is no significant shape difference between the left and right side. Similarly, in our present study, we could not find any difference between the size of the surgery side and non-surgery side of the mandible. The previous analysis did not include surface, cave or F1 hybrids, nor did it analyze the entire jaw shape. Here we have improved the analysis by measuring various dimensions of the mandible and found that the overall shape of the surgery fish mandible more closely resembles the cavefish and F1 hybrid fish mandible shape (Table 2), specifically with respect to the rostral width and side length. A future detailed morphometric study using shape analysis would allow us to understand the effect of lens removal on lower jaw shape more clearly.

## Conclusion

Our study provides a detailed analysis of the oral jaw dentition in surface, cavefish and F1 hybrid fish and shows the close resemblance of F1 hybrids to the cave form. The lens removal (surgery) fish closely resemble the surface morph with respect to the oral dentition. Tooth number, patterning and cuspal morphology have been enhanced in cavefish. This is in contrast to increase in tooth number which was observed on the lens ablated side of the surgery fish. Cavefish also have larger jaws to accommodate these more diverse teeth. Based on our results, we conclude that the molecular and cellular mechanisms which govern the constructive traits in cavefish are different to the mechanisms that cause the increase in tooth number observed in lens-ablated surgery fish.

The morphological resemblance of cavefish and F1 hybrids suggests the dominant characteristics of constructive traits of cavefish. Apart from *Shh*, other signaling pathways, which are upregulated in cavefish evolution, may play a role. Further studies are needed to uncover the possible genetic mechanisms underlying these constructive traits.

## Abbreviations

ANOVA: Analysis of variance; dpf: Days postfertilization; hpf: Hours postfertilization; Shh: Sonic hedgehog.

## Competing interests

The authors declare that they have no competing interests.

## Authors' contributions

ADSA and TFO conceived and designed the experiments. MD stained all the jaws. ADSA and CH analyzed the data. ADSA and TFO wrote the paper. All authors read and approved the final manuscript.

## Supplementary Material

Additional file 1: Figure S1 Bar graphs showing average tooth number on the left and right side of surface, Tinaja cavefish and F1 hybrids and in the surgery side and non-surgery side of surgery fish. Black bar represents the right side of the surface, cave and F1 hybrid fish and the surgery side of the surgery fish. White bar represents the left side of the above three groups and the non-surgery side of the surgery fish mandible. Error bars indicate standard deviation. Click here for file
