# Supplementary material for: Adaptive evolution of the lower jaw dentition in Mexican tetra (Astyanax mexicanus)
Source: EvoDevo. 2013 Oct 7;4:28. doi: 10.1186/2041-9139-4-28 (PMC3852964; doi:10.1186/2041-9139-4-28)
Supplement: Additional file 1: Figure S1 — Bar graphs showing average tooth number on the left and right side of surface, Tinaja cavefish and F1 hybrids and in the surgery side and non-surgery side of surgery fish. Black bar represents the right side of the surface, cave and F1 hybrid fish and the surgery side of the surgery fish. White bar represents the left side of the above three groups and the non-surgery side of the surgery fish mandible. Error bars indicate standard deviation. [file 2041-9139-4-28-S1.pptx]

## Slide 1
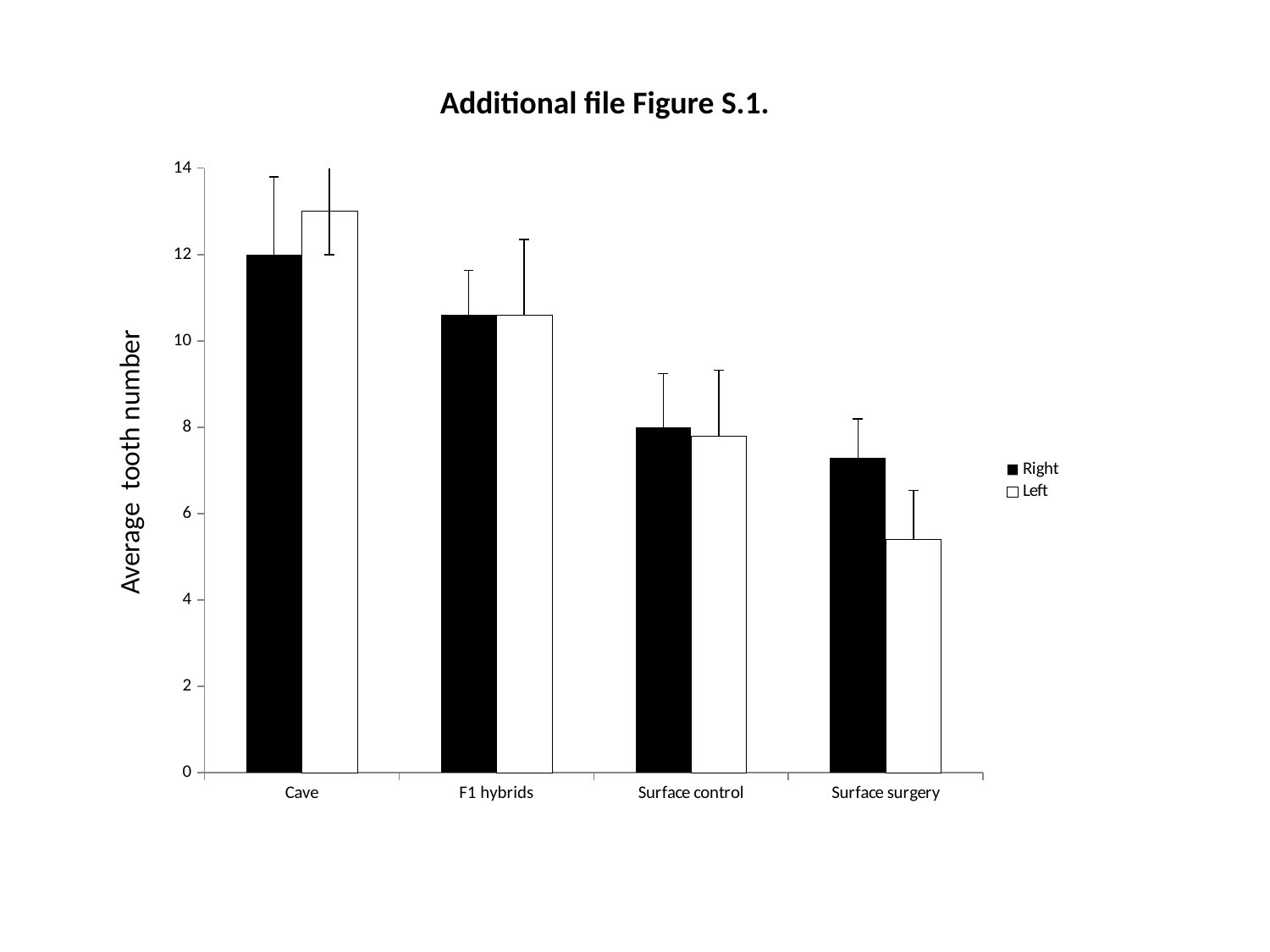

Additional file Figure S.1.
### Chart
| Category | Right | Left |
|---|---|---|
| Cave | 12.0 | 13.0 |
| F1 hybrids | 10.6 | 10.6 |
| Surface control | 8.0 | 7.8 |
| Surface surgery | 7.3 | 5.4 |Average tooth number
